# Pre-PCR Mutation-Enrichment Methods for Liquid Biopsy Applications

**DOI:** 10.3390/cancers14133143

**Published:** 2022-06-27

**Authors:** Farzaneh Darbeheshti, Fangyan Yu, G. Mike Makrigiorgos

**Affiliations:** Department of Radiation Oncology, Dana Farber Cancer Institute and Brigham and Women’s Hospital, Harvard Medical School, Boston, MA 02215, USA; b.darbeheshti70@gmail.com (F.D.); fangyan_yu@dfci.harvard.edu (F.Y.)

**Keywords:** mutation enrichment, pre-PCR enrichment, liquid biopsy, cell-free DNA, circulating tumor DNA, low-level mutation detection

## Abstract

**Simple Summary:**

Liquid biopsies provide a non-invasive approach to tracing tumor-derived biomarkers in blood, with broad applications in medicine. During the past decade, circulating free DNA (cfDNA) has been turned into an informative resource for cancer management. Mutation-enrichment methods enhance the detection of tumor-derived, low-level mutations in blood. These methods increase the frequency of low-level mutations insofar as they become detectable via routine diagnostic techniques. Enriching mutations prior to PCR (pre-PCR) offers distinctive advantages. Applying the mutation-enrichment process directly to genomic DNA or cfDNA circumvents PCR errors and provides enriched mutation-containing products that different technologies can detect without any required changes in their protocols. In this review, we discuss the recent developments in pre-PCR enrichment methods from the perspective of their applications in liquid biopsies.

**Abstract:**

Liquid biopsy is having a remarkable impact on healthcare- and disease-management in the context of personalized medicine. Circulating free DNA (cfDNA) is one of the most instructive liquid-biopsy-based biomarkers and harbors valuable information for diagnostic, predictive, and prognostic purposes. When it comes to cancer, circulating DNA from the tumor (ctDNA) has a wide range of applications, from early cancer detection to the early detection of relapse or drug resistance, and the tracking of the dynamic genomic make-up of tumor cells. However, the detection of ctDNA remains technically challenging, due, in part, to the low frequency of ctDNA among excessive circulating cfDNA originating from normal tissues. During the past three decades, mutation-enrichment methods have emerged to boost sensitivity and enable facile detection of low-level mutations. Although most developed techniques apply mutation enrichment during or following initial PCR, there are a few techniques that allow mutation selection prior to PCR, which provides advantages. Pre-PCR enrichment techniques can be directly applied to genomic DNA and diminish the influence of PCR errors that can take place during amplification. Moreover, they have the capability for high multiplexity and can be followed by established mutation detection and enrichment technologies without changes to their established procedures. The first approaches for pre-PCR enrichment were developed by employing restriction endonucleases directly on genomic DNA in the early 1990s. However, newly developed pre-PCR enrichment methods provide higher sensitivity and versatility. This review describes the available pre-PCR enrichment methods and focuses on the most recently developed techniques (NaME-PrO, UVME, and DEASH/MAESTRO), emphasizing their applications in liquid biopsies.

## 1. Introduction

Over the past decade, liquid biopsies have become an informative complement to tumor tissue biopsies, and provide a minimally invasive approach for repeated interrogation of tumor status. Liquid biopsy encompasses circulating tumor cells (CTCs) and circulating free DNA (cfDNA), including DNA originating from the tumor (ctDNA); circulating free RNA (cfRNA); platelets; and exosomes (EXOs) [1,2]. Among these, circulating tumor cells and circulating tumor DNA (ctDNA) show the greatest potential in cancer medicine at this time [3]. Circulating free DNA, in particular, has growing applications spanning the early detection of minimal residual disease (MRD) [4,5,6,7], early cancer detection in asymptomatic individuals [8,9,10,11,12], the assessment of therapy effectiveness [13,14], and the monitoring of tumor-dynamics [15]. In this perspective, cfDNA can be considered a powerful instrument for detecting early resistance to therapy [16]. This strategy is applicable to various types of cancer, from colon cancer [17,18]—by tracing KRAS mutations in cfDNA following treatment with cetuximab—to lung cancer treated with small molecule inhibitors [19,20], where EGFR resistance mutations are detected. Using COLD-PCR-based mutation enrichment [21,22,23] it was shown that NRAS mutations are an independent prognostic factor for myelodysplastic syndrome [24]. Circulating DNA mutations provide early indications of relapse in melanoma patients undergoing immunotherapy [25]_ENREF_16, and clinical studies indicate the use of cfDNA to complement [26] or replace [27] tissue biopsies. Additional studies validate the use of cfDNA as an informative and specific biomarker for metastatic breast cancer (BC) [28] and its usefulness for the surveillance of remission/relapse [4,6]. In metastatic BC patients, total cfDNA levels are a prognostic and predictive blood-based biomarker associated with progression-free survival, overall survival, and response to treatment [29,30]. The dynamics of circulating tumor DNA (ctDNA) in plasma following initiation of therapy [31] can be prognostic [32], and often, an initial ctDNA rise is followed by a ctDNA decrease within days [33]. The radiation-induced ctDNA dynamic was originally examined using conventional multi-fraction, uniform external-beam radiation therapy [34]. ctDNA dynamics may also depend on single-fraction vs. multi-fraction radiation dose regimens. Additionally, brachytherapy or internal-emitter-based radiation exposure, which deliver non-uniform radiation-induced lethal DNA damage [35,36] to nearby cells [37,38,39] could shape circulating-DNA dynamics differently, both for tumor cells and nearby normal cells.

Additionally, while the clinical significance of low-level mutations of tumor origin in cfDNA is evident, their measurement remains technically challenging. For example, the routine use of next-generation sequencing technology has a lower limit of detection (LOD) for a MAF of ~0.5–1%, below which false-positive signals often emerge due to polymerase mis-incorporations (‘PCR errors’) introduced during sample preparation, and during sequencing-by-synthesis. This unavoidable ‘mutational noise’ is independent of interrogation depth, i.e., increasing the number of reads does not improve the NGS detection limit [5,40,41,42,43]. Enhancements employing computational approaches [5], bi-directional sequencing [44,45], circle sequencing [46], or single-molecule barcoding [40,42,43]_ENREF_3 enable NGS to overcome the noise and detect ‘ultra-rare mutations’. However, these approaches are not straightforward to perform and invariably reduce NGS throughput as they require numerous reads per sequence, while also increasing sequencing cost [47]. One approach to reducing the amount of sequencing needed for the detection of low-level mutations is mutation enrichment prior to sequencing [48]. For example, COLD-PCR mutation enrichment enables targeted resequencing to identify mutations at the 0.02% MAF level with just 28 aligned sequencing reads [41]. Such single-amplicon-based mutation-enrichment approaches have subsequently been applied in cancer [24] and non-invasive prenatal diagnostics [49]. Nevertheless, for effective integration with NGS, highly multiplexed mutation-enrichment approaches are required.

This review focuses on mutation-enrichment approaches that can be applied prior to performing a PCR step (Table 1). Such pre-PCR mutation-enrichment approaches have certain advantages. They take place at the genomic DNA level, thereby bypassing the influence of PCR-introduced mis-incorporations that produce false-positives. They can often be highly multiplexed. Additionally, they are versatile, as they can often be seamlessly combined with established amplification and endpoint detection methods including isothermal amplification [50], NGS, digital PCR [51], real time PCR [52] and high-resolution melting [53]. We focus on recently developed pre-PCR mutation-enrichment methods (NaME-PrO, UVME) as well as on recent enhancements of earlier methods (DEASH, MAESTRO).

## 2. NaME-PrO

**Figure 1:**
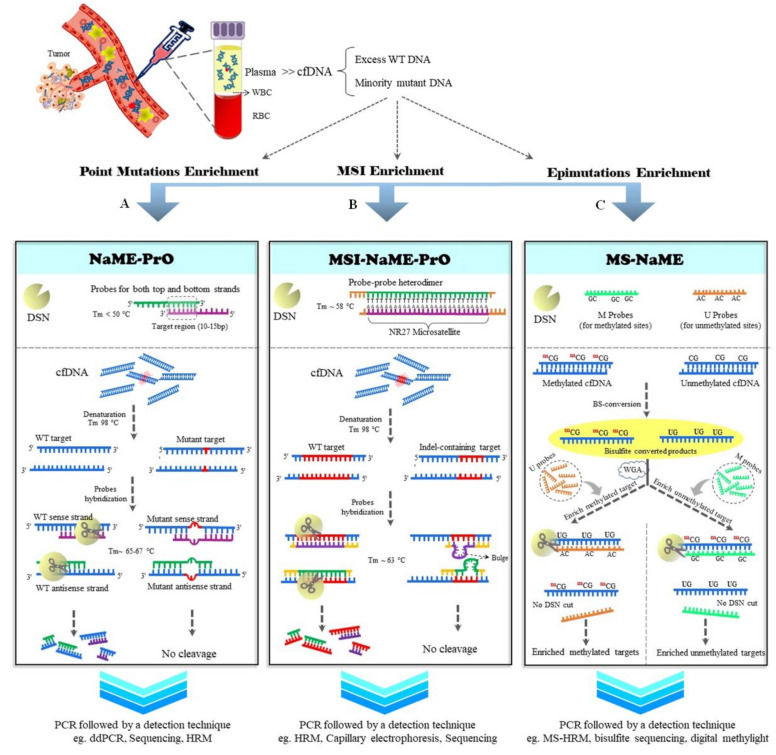
Applications of nuclease-assisted minor-allele enrichment (NaME) method in cell-free DNA (cfDNA) or genomic DNA: (**A**) Workflow for NaME using probe overlap (NaME-PrO) for enrichment of mutations. The overlapping probes are designed to match sense and antisense strands of wild-type (WT) targets. The nucleotides within the probe overlap region determine the target sequence. Following denaturation step and probe hybridization, duplex-specific nuclease (DSN) preferentially digests fully matched double-stranded sequences, thereby retaining intact the mutated strands; (**B**) workflow for enrichment of indel-containing sequences to enhance detection of microsatellite instability (MSI-NaME-PrO). The overlapping probes for targeting microsatellite NR27, as an example, are illustrated. The polyA homopolymer has a low Tm, such that the probe–probe and the probe–target hybrids show a ~5 °C difference. Following probe hybridization, DSN digests WT alleles, while indel-containing sequences remain substantially undigested because of bulges within probe–target hybrids; (**C**) workflow of methylation-sensitive NaME (MS-NaME): U probes are designed to bind uracil-containing sequences (which are generated via bisulfite-mediated cytosine deamination). M probes are designed to bind 5mC-containing DNA. Following denaturation and whole-genome amplification (WGA), either U probes or M probes are applied to enrich methylated and unmethylated targets, respectively.

Nuclease-assisted minor-allele enrichment with probe-overlap (NaME-PrO) is a scalable enrichment technique where the genotypic selection step is applied directly on genomic DNA or cfDNA without pre-amplification [54]. After DNA denaturation (Figure 1A), the temperature is lowered, and target-specific overlapping probes are employed to bind the sense and antisense WT strands. Subsequently, fully complementary target–probe hybrids are digested efficiently by double-strand-DNA-specific nuclease DSN [55], while mutation-containing strands are protected from enzymatic digestion due to mismatches within mutant DNA–probe hybrids and due to reduced duplex formation with the probes. The short oligonucleotide probes (20–25 bp) are designed as follows: Target–probe hybrids have Tm of 63–67 °C for wtDNA. There is a 10–15 bp overlapping region between the top and bottom probes, such that probe–probe hybrids have a Tm less than 50 °C; this region determines the target sequence for enrichment. The 3′ end of the probes can be blocked to inhibit polymerase extension in the subsequent amplification step. NaME-PrO enables the detection of low-level mutations at frequencies of ~0.01%, which also depends on the sequence context and endpoint detection method, in a pre-PCR reaction [54]. NaME-PrO was originally applied for enriching cancer-specific mutations in cfDNA samples using ddPCR or Illumina MiSeq sequencing as endpoint-detection techniques [54]. It can also be used for SNP genotyping in cfDNA samples, with potential application in tracking specific SNPs in recipient cfDNA following solid-organ transplantation [54,56]. In multiplexed application, NaME-PrO was used in a 54-plex reaction to investigate the “tumor fingerprint” in cfDNA from breast cancer patients. This approach could identify at least one tumor-specific mutation at a frequency <0.01% in cfDNA without requiring deep sequencing [57].

To avoid protocols that require the opening of PCR vials and the addition of probes and DSN following the initial DNA denaturation, a modified closed-tube approach called no-denaturation nuclease-assisted minor-allele enrichment with probe-overlap (ND-NaME-PrO) was also developed [58]. This approach enables the addition of probes and DSN at 4 °C followed by the application of NaME-PrO at 65 °C, thus avoiding inactivation of the enzyme by denaturation at 95 °C. Selective digestion of WT sequences still occurs in this protocol, potentially due to DNA breathing at 65 °C [59] which allows probes to transiently bind their respective targets, and leads DSN to cleave fully matched WT strands [58]. Although some mutated dsDNA is also digested by DSN during ND-NaME-PrO, the mutant digestion efficiency is significantly lower than that of the fully matched DNA–probe hybrids. ND-NaME-PrO was validated in genomic DNA and cfDNA from clinical samples, followed by HRM, ddPCR, and MiSeq targeted resequencing as endpoint diagnostic tools [58]. During mutation analysis in cfDNA from metastatic breast cancer patients, the multiplexed application of ND-NaME-PrO could lead to a ~25-fold average enrichment in targeted mutations. The data indicate that the combination of ND-NaME-PrO with HRM can be a practical pre-screening test to track minimal residual disease or early detection in liquid biopsies. In view of partial digestion of mutated sequences along with WT DNA, a pre-amplification step is recommended before ND-NaME-PrO.

The application of NaME-PrO directly on genomic DNA/cfDNA prior to PCR enables straightforward combination with established mutation-enrichment technologies operating during PCR amplification. The combination of NaME-PrO with the Amplification Refractory Mutation System (ARMS)—called a NAPA assay—boosts the analytical specificity of ARMS [60]. ARMS-PCR is one the most common methods for point-mutation detection, but can occasionally produce false-positives because of non-specific priming, particularly in the case of low-level mutations. Markou et al. [60] showed that NAPA removes this limitation when applied to the detection of hotspot *PIK3CA* mutations (H1047R and E545K) in cfDNA and circulating tumor cells (CTCs) from metastatic BC patients. *PIK3CA* is one of the frequently mutated genes in different cancers with valuable predictive potential [61,62]. NAPA could simultaneously detect the minority mutant alleles within *PIK3CA* at a frequency of 0.1–0.5% with high specificity. The NAPA assay was similarly applied to detect hotspot *ESR1* mutations in both CTC and cfDNA samples from BC patients with improved sensitivity and specificity [63].

Keraite et al. [64] applied the NaME-PrO method to simultaneously enrich four hotspot mutations of the *PIK3CA* gene (E545K, E542K, H1047L, and H1047R) in cfDNA from BC samples. This study applied NaME-PrO following a target pre-amplification to accurately detect *PIK3CA* mutations in cfDNA via real-time qPCR or ddPCR. Using SYBR Green real-time qPCR makes this approach accessible and affordable for many laboratories. Moreover, a simple prediction approach has been introduced to determine the initial mutation frequency in blood samples using a logarithmic regression method. This approach increases the mutant allele frequency up to 30-fold, improves detection sensitivity, and provides close-to-quantitative results, which is important for the clinical interpretation of cfDNA samples.

### 2.1. MSI-NaME-PrO

Microsatellite instability (MSI) refers to frequent indels within repetitive genomic sequences, resulting from deficiencies in cellular DNA mismatch repair [65]. Tracing MSI in cancer patients has been accentuated because of its roles in diagnosis, prognosis, and personalized therapy [66]. Sensitive MSI detection methods via liquid biopsies are important for the genotyping of primary or secondary tumors when a tissue biopsy is not clinically available. MSI-NaME-PrO, a modification of NaME-PrO, is a pre-PCR enrichment method that traces MSI (Figure 1B). MSI-NaME-PrO removes unaltered micro-satellite targets using probes for multiple microsatellite sites simultaneously. Since MSI within target sequences induces ‘bulges’ between the probe–target hybrids, DSN does not efficiently digest target sequences containing microsatellite indels. Accordingly, MSI-NaME-PrO reduces false-positive results derived from polymerase slippage in homopolymer sequences, and improves indel detection sensitivity [67]. The data indicate the high sensitivity and specificity of combining MSI-NaME-PrO and capillary electrophoresis for detecting MSI at a frequency of ~0.01% in cfDNA samples from colorectal cancer patients [67].

Another modification of NaME-PrO for enriching MSI prior to PCR amplification is nuclease-assisted microsatellite instability enrichment (NaMSIE) [68]. NaMSIE employs two overlapping LNA probes complementary to the WT allele of microsatellite HT17. Consequently, DSN digests WT templates, and enriches indel-containing alleles. The enriched products undergo PCR and capillary electrophoresis. NaMSIE method provides the detection of MSI with an LOD of 0.5%.

### 2.2. MS-NaME

Tracing aberrant methylation signatures as “epimutations” in liquid biopsies can boost the utility and clinical value of cfDNA in different diseases, including cancer [69,70,71]. Another adaptation of NaME-PrO technology, methylation-specific nuclease assisted minor-allele enrichment (MS-NaME), has been applied for enriching methylated/unmethylated targets from genomic DNA/cfDNA [72]. MS-NaME uses probes to lead DSN to multiple targets following sodium bisulfite treatment of DNA (Figure 1C). Bisulfite converts unmethylated cytosine residues to uracil, while 5-methylcytosines (5mC) remain unaltered [73]. Depending on the probe sequences, it is possible to enrich either methylated targets or unmethylated targets in bisulfite-treated DNA. If the probes are designed to match uracil-containing sequences (U-probes)—generated via bisulfite-mediated cytosine deamination—the unmethylated DNA is digested by DSN, thereby enriching the 5mC-containing targets. In the reverse approach, it is possible to rescue unmethylated DNA from DSN digestion, if the probes are designed to bind 5mC-containing DNA (M probes). As a result, unmethylated targets are enriched. This technique can be applied directly to bisulfite-converted genomic DNA. Alternatively, it is possible to apply whole-genome amplification (WGA) of bisulfite-treated DNA prior to MS-NaME since bisulfite treatment results in significant DNA degradation and cfDNA is usually of limited quantity. Liu et al. [72] applied U-probes to enrich methylated minority alleles in cfDNA samples in a 177-plex setting, followed by MS-HRM or MS-TaqMan-based digital PCR. This method could detect 1% methylation in ATM gene promoters [72].

## 3. UVME

**Figure 2:**
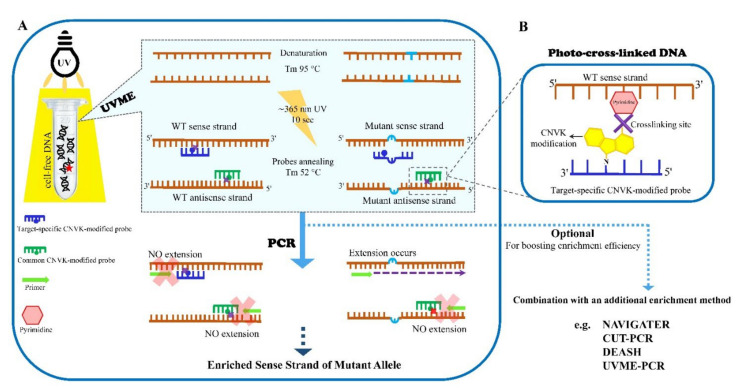
Workflow of pre-PCR-UV-mediated cross-linking minor-allele enrichment (pre-PCR-UVME): (**A**) A UV lamp is employed to induce selective cross-linking of wtDNA. Target-specific CNVK-modified probes bind sense strand of wtDNA, and common CNVK-modified probes attach to antisense strands of both wtDNA and mutated DNA; (**B**) after UV irradiation, fully matched probe–target hybrids undergo photo-crosslinking of CNVK with C or T on the target sequence, thereby inhibiting the proliferation of wtDNA strands and mutant antisense strand during PCR. Mismatch-containing probes undergo significantly lower crosslinking.

UV-mediated cross-linking minor-allele enrichment (UVME) is a newly developed technique for pre-PCR mutation enrichment that utilizes UV irradiation to block wtDNA from subsequent amplification [74]. The UVME principle (Figure 2A) lies in using 3-cyanovinylcarbazole nucleoside (CNVK)-modified probes matching the wild-type DNA sequence. Upon UV irradiation at 365 nm, CNVK induces photo-cross-linking with pyrimidines on the opposite DNA strand (Figure 2B) [75,76]. The two probes are designed for: UVME, a target-specific probe matching the sense strand of WT target sequences and creating mismatches with mutated sequences; and probes that bind the antisense strands of both wtDNA and mutated DNA, thereby blocking the amplification of both WT and mutant antisense strands. Following DNA denaturation and probe hybridization, 10 s UV irradiation is applied to induce cross-links between CNVK-modified probes and a pyrimidine within the target sequence. Mismatch-containing oligonucleotide probes at mutation positions undergo significantly less crosslinking, thereby leading to mutation enrichment during subsequent amplification.

UVME can operate in two different settings, pre-PCR-UVME and UVME-PCR. The former (Figure 2) is directly applied to fragmented genomic DNA or cfDNA. Pre-PCR-UVME applies the genotypic selection step prior to PCR, reducing the influence of PCR errors, and can be followed by existing detection techniques without any change in their established procedure. It is also amenable to employing different CNVK-modified probes against multiple targets. In the application of pre-PCR-UVME in liquid biopsies, plasma cfDNA from breast cancer patients with confirmed mutations was used. The pre-PCR-UVME enrichment method could increase the initial mutation allelic frequency ~30-fold. This enrichment capacity improves the nominal limit of detection of multiple detection technologies, such as NGS, Sanger, and HRM, and also enhances ddPCR signals for detecting low-level mutations [74].

The latter approach, UVME-PCR, employs UV irradiation during each PCR cycle. Following 10 cycles pre-amplification without UV, a second PCR stage is initiated, that blocks wtDNA at the primer annealing temperature of each PCR cycle, resulting in robust mutation enrichment. UVME-PCR technically shows higher enrichment efficiency in comparison to pre-PCR-UVME. However, pre-PCR UVME is easier to perform, using a standard UV lamp and a PCR machine or a simple heating block. UVME-PCR, on the other hand, employs UV at each PCR step. The use of an external UV lamp manually during PCR annealing steps, as carried for demonstrating the UVME principle, is laborious. Accordingly, incorporating a UV source within PCR instruments is imperative if UVME-PCR is to be utilized broadly. The combination of pre-PCR-UVME with UVME-PCR shows remarkable mutation enrichment, which converts the original mutation frequencies of 0.1% and 1% to 85% and 98%, respectively [74].

## 4. DEASH

**Figure 3:**
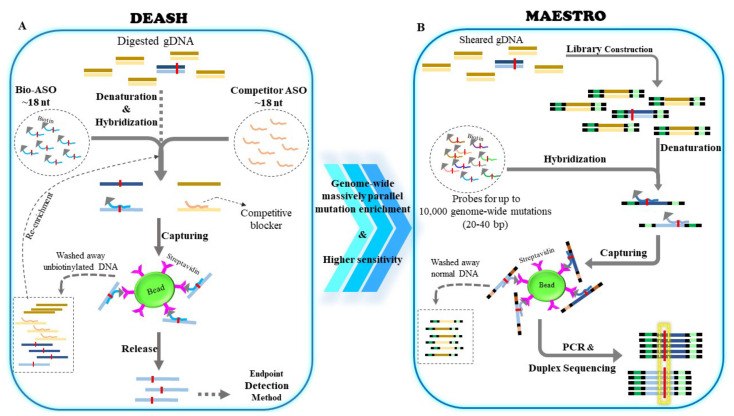
(**A**) Principles of DNA enrichment via allele-specific hybridization (DEASH), and minor-allele-enriched sequencing through recognition oligonucleotides (MAESTRO). The biotinylated allele-specific oligonucleotide (bio-ASO) is designed to bind mutant strands, while competitor ASO attaches to wild-type strands. Following denaturation and hybridization, the bio-ASO/target hybrids are captured by streptavidin-coated beads. The unbound DNA can undergo further cycles of DEASH. The enriched single-stranded targets are eluted and purified; (**B**) workflow for minor-allele-enriched sequencing through recognition oligonucleotides (MAESTRO) technique. The underlying principle is similar to DEASH, although MAESTRO operates without competitive blockers and provides genome-wide massively parallel enrichment. Following NGS library construction and barcoding top and bottom DNA strands, the biotinylated probes complementary for up to 10,000 genome-wide mutations are employed to bind mutant strands. Afterwards, the mutation-containing strands are preferentially captured by streptavidin-coated beads, are amplified, and undergo duplex sequencing.

An alternative approach for mutation enrichment is the selective capturing of mutant alleles on a solid support instead of removing wtDNA through enzymatic reactions or UV-mediated cross-linking. In 2003, Jeffreys et al. [77] described a bead-based capturing technique for enriching known single-nucleotide variants via biotinylated allele-specific oligonucleotides. DNA enrichment using the allele-specific hybridization (DEASH) method can increase the frequency of mutant alleles prior to PCR (Figure 3A). DEASH is applied on restriction-endonuclease-digested dsDNA. Biotinylated allele-specific oligonucleotides (bio-ASOs) are employed to bind mutant alleles. The competitor ASO complementary to wtDNA, without a biotin tag, is also simultaneously used to improve the hybridization specificity. Following hybridization, the bio-ASO/target hybrids are captured using streptavidin-coated magnetic beads. Purified captured products can undergo further rounds of DEASH. This study reported that rare mutations of mutation allelic frequency ~10^−3^–10^−5^% could become detectable by applying DEASH prior to amplification. Although the originally described DEASH method was not applied on cfDNA samples, a recent development uses a modified DEASH principle to track thousands of low-level mutations in cfDNA. Minor-allele-enriched sequencing through recognition oligonucleotides (MAESTRO), employs short probes to capture up to 10,000 low-level mutations simultaneously, and then, incorporates duplex sequencing to detect mutation fingerprints in cfDNA for tracking minimal residual disease with high sensitivity and specificity (Figure 3B) [78].

## 5. Additional Methods for Mutation Enrichment Directly from Genomic DNA

While the principal modus operandi for NaME-PrO, UVME, and DEASH involves direct application to genomic DNA, there are a number of mutation-enrichment methods that may, in principle, also be applied directly to genomic DNA or cfDNA. To provide a comprehensive view, we briefly discuss these methods.

### 5.1. Restriction-Enzyme-Mediated Enrichment

Restriction endonucleases (REs) cleave DNA strands through a specific recognition site [79]. These enzymes have broad application in biology, such as gene cloning, DNA mapping, and mutation detection [80]. Due to their digestion fidelity and affordability, pre-PCR mutation enrichment has benefited from various types of RE [48]. A limitation of using REs is that they can only be applied to mutations that alter their specific recognition sequence.

#### 5.1.1. Restriction-Site Mutation (RSM) and Restriction-Fragment-Length Polymorphism (RFLP)-PCR

In 1990 Parry et al. proposed the RSM technique for detecting mutations in restriction-enzyme recognition sites [81]. Although initial experiments using RSM focused on studying mutagen-induced mutations in vitro, it subsequently evolved to a method to detect rare clinical mutations [82]. This sensitive enrichment method is based on the resistance of mutated DNA to endonuclease activity. Any base change within the restriction-enzyme recognition site results in losing the catalytic activity of a specific RE. Consequently, following incubation with the particular RE under optimal conditions, the resistant and intact mutated DNA strands could be amplified using PCR. Initial RSM investigations have indicated mutation detection at a 10^–3^ frequency, while improvements in digestion efficiency have led to higher sensitivity up to 10^−6^. RSM is simple, cost-effective, and sensitive; however, the method is subject to false positives when enzymatic digestion is incomplete or when the sample contains impurities that inhibit the enzyme. RSM should be optimized carefully for a specific sequence and enzyme combination. In 1992, Sandy et al. [83] developed a similar approach, RFLP-PCR. According to their results, this method could enrich ten mutant alleles among 10^8^ to 10^9^ WT strands. However, this sensitivity comes at a cost. There is a preparative step of restriction digestion to enrich the target sequence from genomic DNA. In the next step, the main digestion process with specific RE is performed, and undigested targets, including mutations, are amplified. The PCR products are cloned and detected by reporter probes [84]. The above-mentioned approaches could theoretically be applied to any known point mutations that modify the recognition site conferring resistance to cleavage. The potential for false positives resulting from impurity-impaired enzymatic digestion and the requirement for mutations that alter the restriction sites comprise the main drawbacks of RE-based assays.

#### 5.1.2. Random Mutation Capture (RMC)

Random Mutation Capture assay is a derivation of the RSM technique that incorporates restriction-enzyme-mediated enrichment and bead-based capture [85,86]. Random mutations may occur in a small minority of the cell population, especially in cases of mismatch repair deficiency [87]. The RMC technique applies uracil-containing biotinylated probes to capture the target sequence from extensive digested genomic DNA with REs that do not cleave the target sequence. This step improves the digestion efficiency for digesting wild-type targets. The hybridized targets are captured by streptavidin magnetic beads and undergo five rounds of “hybridization-digestion” with a specific RE. In this step, WT strands are cut by RE, while any mutation within the enzyme recognition site hampers RE digestion. Finally, the probes are digested by uracil-DNA glycosylase. The uncut sequence harboring a mutation at the RE recognition site is detected via real-time qPCR using primers that flank the RE recognition sequence. RMC renders an enrichment method with extremely high sensitivity (1 mutant allele per 10^8^ WT alleles). It also provides simultaneous and quantitative analysis of mutations in multiple genes. This method has been used to investigate random point-mutations in normal and malignant human tissues [88]. The results indicated a high frequency of spontaneous random mutations in certain tumors. However, there are no data on whether this technique can be adapted for use with cfDNA.

### 5.2. CUT-PCR

CRISPR-based mutation-enrichment approaches have emerged over the past decade [89,90,91]. They benefit from the simplicity, affordability, and accuracy of CRISPR-Cas nucleases. Various mutation-enrichment methods have been developed using different Cas enzymes and their specific catalytic activity properties. However, most of them are hampered in their widespread application by their dependency on a specific motif within the target sequence called a protospacer adjacent motif (PAM). CRISPR-mediated ultrasensitive detection of target DNA-PCR (CUT-PCR) can efficiently enrich rare mutant alleles by depleting unwanted wtDNAs before PCR amplification [92]. In this approach, the Cas9 endonuclease uses single-strand guide RNAs specific to PAM containing wtDNA, thereby selectively removing high-abundance WT sequences. Using CUT-PCR, five frequent KRAS mutations (c.34G4C, c.35G4C, c.35G4A, c.34G4T, and c.35G4T) in cfDNAs from colorectal cancer patients were analyzed [92]. This study used the type-II CRISPR endonuclease Cas9, termed SpCas9. The five mutations could be enriched by a common guide RNA, because all of them are within the PAM sequence and obstruct SpCas9 activity. The results demonstrated that the frequency of mutant alleles increases up to 600-fold relative to the frequency of WT alleles, after three rounds of CUT-PCR. According to the authors and the COSMIC database, this method could be effective on 80% of known cancer-related point mutations by employing a variety of orthogonal Cas enzymes. The CUT-PCR technique could potentially enrich rare mutations at a mutation allelic frequency as low as 0.01% with high accuracy, comparable with targeted deep sequencing. By engineering the available Cas endonucleases and modifying their required PAM sequence, the utility of this technique could potentially be expanded. Wang et al. applied a similar approach to enrich EGFR exon19 deletion mutations in cfDNA from non-small-cell lung cancer patients [93]. They employed a pre-amplification step to optimize CRISPR/Cas9 enrichment. The results revealed about a 1000-fold increase in mutant allele frequency in comparison with the samples without enrichment.

### 5.3. NAVIGATER

The nucleic acid enrichment via DNA-Guided Argonaute from *Thermus thermophilus* (NAVIGATER) technique is based on nucleic acid-guided endonuclease activity of the TtAgo enzyme to efficiently cleave DNA targets complementary to its single-strand guide DNA (gDNA) [94]. TtAgo is a multi-turnover enzyme that does not require a specific motif for cleaving its targets. This assay employs small gDNAs, optimally 15-16nt, that are designed to bind sense and antisense strands of target wtDNA completely. Any single-nucleotide mismatch between the gDNA and the target region hinders the cleavage activity of TtAgo. An interesting point about the NAVIGATER method is that there is no separate denaturation step in the enrichment process. Due to TtAgo’s activity at high temperatures, the gDNA hybridization and WT cleavage procedures happen in a one-hour incubation time at ~80 °C. The dsDNAs unwind when the incubation temperature is increased, and gDNAs have to chance to bind their targets. Song et al. [94] applied NAVIGATER on cfDNA samples from pancreatic cancer patients after 35 cycles of pre-amplification for *KRAS* G12D*/*V*/*R mutations. The results revealed that twice-NAVIGATER, followed by xenonucleic acid clamp PCR (XNA-PC), could detect rare mutations at a mutation allelic frequency <0.2%, with a specificity comparable to tissue NGS genotyping. The authors also applied NAVIGATER directly to genomic DNA and cfDNA without pre-amplification to track *KRAS* G12D mutant alleles. The results indicate that this method could potentially enrich cell-free mutant alleles without pre-amplification with acceptable efficiency, since NAVIGATER can operate with short size DNA strands, such as those in cfDNA ~160 bp, or formalin-degraded samples [95]. Nevertheless, removing the pre-amplification step from the NAVIGATER protocol results in reduced mutation-enrichment efficiency [94].

### 5.4. PI Polyamides

Pyrrole-imidazole (PI) polyamide bead-based capture may also potentially enrich rare specific mutant alleles in cfDNA samples in a pre-PCR setting. By employing the affinity of PI polyamides to attach to a particular sequence of the minor groove of double helix B-DNA, the hotspot KRAS mutations in cfDNA samples are successfully captured and detected by ddPCR [96]. For this purpose, specific biotinylated PI polyamides are designed and synthesized to target KRAS codon 12 mutations, such that they bind the target mutation sequence with high affinity. The biotinylated PI polyamides are attached to streptavidin magnetic beads and incubated with cfDNA for one hour. After washing to remove wtDNA, the attached mutant strands are eluted and retrieved. Applying this method for non-metastatic colorectal cancer patients demonstrates a remarkably increased sensitivity of ctDNA analysis compared with using ddPCR without PI polyamide enrichment (88.9% vs. 11.1%). PI polyamide bead-based enrichment could boost the frequency of the target mutations up to 27-fold for the initial frequency of 0.1. On the other hand, it does not provide a versatile and multiplex enrichment method due to PI polyamides’ affinity to the requirement for specific sequences and a specific structure of DNA for enrichment to occur.

## 6. Discussion and Conclusions

Liquid biopsy harbors valuable information for the clinical management of disease and has become a hot topic in cancer research and diagnostics. cfDNA is an informative molecular biomarker that can be tracked to detect tumor-related mutations, or other DNA alterations. Notable advancements in mutation-enrichment methods provide the ability to increase the frequency of low-level mutations in liquid biopsies so that they become detectable by commonly used detection techniques. Among all enrichment techniques, pre-PCR enrichment methods offer distinct advantages such as the avoidance of PCR errors. Polymerase mis-incorporations take place whenever DNA is amplified, and the rate of mis-incorporation depends on the proof-reading ability of the polymerase used, as well as the sequence context [97,98]. PCR errors become more evident when methods that screen all sequence positions are applied following amplification, such as NGS, as in such cases, it is more probable that hotspots for base mis-incorporation will be revealed. Applying a genotypic selection step directly to genomic DNA or cfDNA prior to PCR not only circumvents PCR errors, but provides enriched products that can be detected by various endpoint detection technologies. As sequencing costs continue to decrease, mutation enrichment may provide less of a cost advantage. Yet, NGS methods such as duplex sequencing need an ultra-high sequencing depth to provide high accuracy. This restriction becomes more limiting as the targeted mutation panel is enlarged. Mutation-enrichment methods provide a unique solution in this case. To this end, an improved version of the DEASH method, termed MAESTRO [78], provides a feasible enrichment approach for large target duplex sequencing at low cost, thus providing both ‘breadth and depth’. On the other hand, the NaME-PrO and pre-PCR-UVME methods provide multi-target enrichment principles that could potentially be applied to cfDNA directly and used with any downstream method, including NGS. They are promising approaches to tracking multiple low-level mutations in liquid biopsies in routine clinical practice. Overall, pre-PCR enrichment methods comprise an achievable, simple, and affordable approach to tracing informative mutations in circulating DNA. Beyond cancer management, mutation-enrichment methods can have applications in non-invasive prenatal testing (NIPT). For example, for single-gene disorders, the detection of paternally inherited mutations in cell-free fetal DNA can be sensitively traced within excess maternal cfDNA following mutation enrichment [49]. Increasing the sensitivity for fetal DNA detection could potentially lead to prenatal diagnosis earlier in pregnancy. In perspective, while the applications of circulating DNA as a biomarker grow, so does the need for improved technologies to overcome the formidable technical difficulties associated with reliable biomarker detection. Continued technological improvement can be anticipated in this field.

## Figures and Tables

**Table 1:** Pre-PCR mutation-enrichment techniques for liquid biopsy. LOD—limit of detection; MAF—mutant allelic fraction.

Method	Operation	Target Motif Requirement	Mutation Type	LOD (%MAF)	Multiplexity	Adjustment for Liquid Biopsy
NaME-PrO ^1^	Specific probes and duplex-specific nuclease	NO	Point Mutations	10^−2^	Moderate	YES
MSI-NaME-PrO ^2^	Specific probes and duplex-specific nuclease	NO	Microsatellite instability	10^−2^	Low	YES
NaMSIE ^3^	LNA probes and duplex-specific nuclease	NO	Microsatellite instability	5 × 10^−1^	Low	NO
MS-NaME ^4^	Specific probes and duplex-specific nuclease	NO	Epimutations	10^−2^	Moderate	YES
Pre-PCR-UVME ^5^	Specific probes and UV irradiation	NO	Point Mutations	10^−2^	High	YES
DEASH ^6^	Biotinylated allele-specific oligonucleotides and magnetic bead capture	NO	Point Mutations	10^−3^–10^−5^	Low	NO
MAESTRO ^7^	NGS library construction, biotinylated allele-specific oligonucleotides, magnetic bead capture, and duplex sequencing	NO	Point Mutations	10^−3^	High	YES
RSM ^8^	Restriction endonuclease	YES	Point Mutations	10^−1^–10^−4^	Low	NO
RFLP-PCR ^9^	Restriction endonuclease	YES	Point Mutations	10^−5^	Low	NO
RMC ^10^	Magnetic bead capture and restriction endonuclease	YES	Point Mutations	10^−1^–10^−6^	Low	NO
CUT-PCR ^11^	CRISPR-Cas9 and guide RNA	YES	Point Mutations	10^−2^	Low	YES
NAVIGATER ^12^	TtAgo endonuclease and guide DNA	NO	Point Mutations	10^−1^–10^−2^	Moderate	YES
PI Polyamides ^13^	Biotinylated PI polyamides and magnetic bead capture	YES	Point Mutations	10^−1^	Low	YES

^1^ Nuclease-assisted minor-allele enrichment assay with overlapping probes; ^2^ microsatellite-instability nuclease-assisted minor-allele enrichment assay with overlapping probes; ^3^ nuclease-assisted microsatellite instability enrichment; ^4^ methylation-specific nuclease-assisted minor-allele enrichment; ^5^ pre-PCR UV-mediated cross-linking minor-allele enrichment; ^6^ DNA enrichment via allele-specific hybridization; ^7^ minor-allele-enriched sequencing through recognition oligonucleotides; ^8^ restriction-site mutation; ^9^ restriction-fragment-length polymorphism-PCR; ^10^ random mutation capture; ^11^ CRISPR-mediated, ultrasensitive detection of target-PCR; ^12^ nucleic acids of clinical interest via DNA-guided Argonaute from Thermus thermophilus; ^13^ pyrrole-imidazole polyamides.
